# Annual Report of the National Board of Health for the Year 1885

**Published:** 1886-06

**Authors:** 


**Annual Report of the National Board of Health for the year 1885.** Washington: Government Printing Office. 1886.

This report contains a great deal of information of value to the profession, and our readers should apply promptly to the representative of their district in Congress, or to the Senator for their State, from whom they can obtain a copy on application.

